# Tailoring the monomers to overcome the shortcomings of current dental resin composites – review

**DOI:** 10.1080/26415275.2023.2191621

**Published:** 2023-04-20

**Authors:** Jingwei He, Lippo Lassila, Sufyan Garoushi, Pekka Vallittu

**Affiliations:** a College of Materials Science and Engineering, South China University of Technology, Guangzhou, China; b Department of Biomaterials Science and Turku Clinical Biomaterials Center-TCBC, Institute of Dentistry, University of Turku, Turku, Finland; c Wellbeing Services County of South-West Finland, Turku, Finland

**Keywords:** Dental resin composites, monomers, polymerization shrinkage, antibacterial activity, radio-opacity, Bis-GMA free

## Abstract

Dental resin composites (DRCs) have become the first choice among different restorative materials for direct anterior and posterior restorations in the clinic. Though the properties of DRCs have been improved greatly in recent years, they still have several shortcomings, such as volumetric shrinkage and shrinkage stress, biofilm development, lack of radio-opacity for some specific DRCs, and estrogenicity, which need to be overcome. The resin matrix, composed of different monomers, constitutes the continuous phase and determine the performance of DRCs. Thus, the chemical structure of the monomers plays an important role in modifying the properties of DRCs. Numerous researchers have taken to design and develop novel monomers with specific functions for the purpose of fulfilling the needs in dentistry. In this review, the development of monomers in DRCs were highlighted, especially focusing on strategies aimed at reducing volumetric shrinkage and shrinkage stress, endowing bacteriocidal and antibacterial adhesion activities as well as protein-repelling activity, increasing radio-opacity, and replacing Bis-GMA. The influences of these novel monomers on the properties of DRCs were also discussed.

## Introduction

Dental resin composites (DRCs) are esthetic restorative materials composed of the resin matrix, inorganic fillers, and coupling agents. Due to their versatility, DRCs have several applications in dentistry, such as restorative materials, cavity liners, pit and fissure sealants, luting materials, endodontic sealers, and root canal posts [1]. With the problems that exist in the clinic and the requirements from clinicians, DRCs still face several challenges regarding the incidence of failed composite restorations, secondary caries, toxicity, etc., even though the properties of DRCs have been improved markedly in recent years [2]. The modification of DRC composition can be approached through tailoring the resin matrix [3–6], the fillers [7,8], and/or the coupling agents [9–11]. Modification of resin matrix has often seemed to be most effective e.g. when attempting to reduce polymerization shrinkage stress [12].

The resin matrix is a mixture of different monomers containing a small amount of an initiation system. After light or heat activation, monomers can polymerize in a short time to form a cross-linked polymeric network constituting a continuous phase which binds the fillers together [13]. Macroscopically, the DRCs transform from a liquid or paste state into a solid state, which can withstand a certain load. The composition and chemical structure of the monomers significantly influence the properties of the polymer, thereby determining the performance of the cured DRCs. Therefore, the design and synthesis of novel monomers for the purpose of fulfilling the needs of dentistry is one of the research hotspots in dental materials science.

Usually, monomers used in thermoset DRCs are di(methyl)acrylate compounds, but in the last two decades, several monomers with other kinds of polymerizable functional groups were introduced into DRCs. This review will focus on the development in the design of monomers for overcoming the drawbacks of DRCs, and the influences of these novel monomers on the properties of DRCs will also be discussed.

## Monomers for reducing volumetric shrinkage and shrinkage stress

During the polymerization of cross-linkable thermoset DRCs, weak van der Waals interaction between monomers is replaced by strong covalent bonds, leading to the reduced distance between monomers and thus to volume shrinkage [14–17]. As a critical drawback of DRCs, volumetric shrinkage and related stress on the adjacent tooth structure is a major concern in restorative dentistry [18]. After being applied and light-cured in the prepared cavity, the volumetric shrinkage and elastic modulus of DRC develop and increase gradually during polymerization, but shrinkage stress can be relieved by the rearrangement of the polymer in the early stage of polymerization. At some stage, the gradual development and increase in elastic modulus hinder any further adsorption of stress caused by the volumetric shrinkage and lead to stress being generated within the material, at the tooth/restoration interface and in the tooth structure [19]. If the shrinkage stress is sufficiently concentrated, it may induce interfacial bond failure, microleakage, post-operative sensitivity, deformation of the tooth cusps, marginal staining, and the formation of secondary caries [1,12,17,20–22].

### Low volumetric shrinkage methacrylates

**Figure 1.**
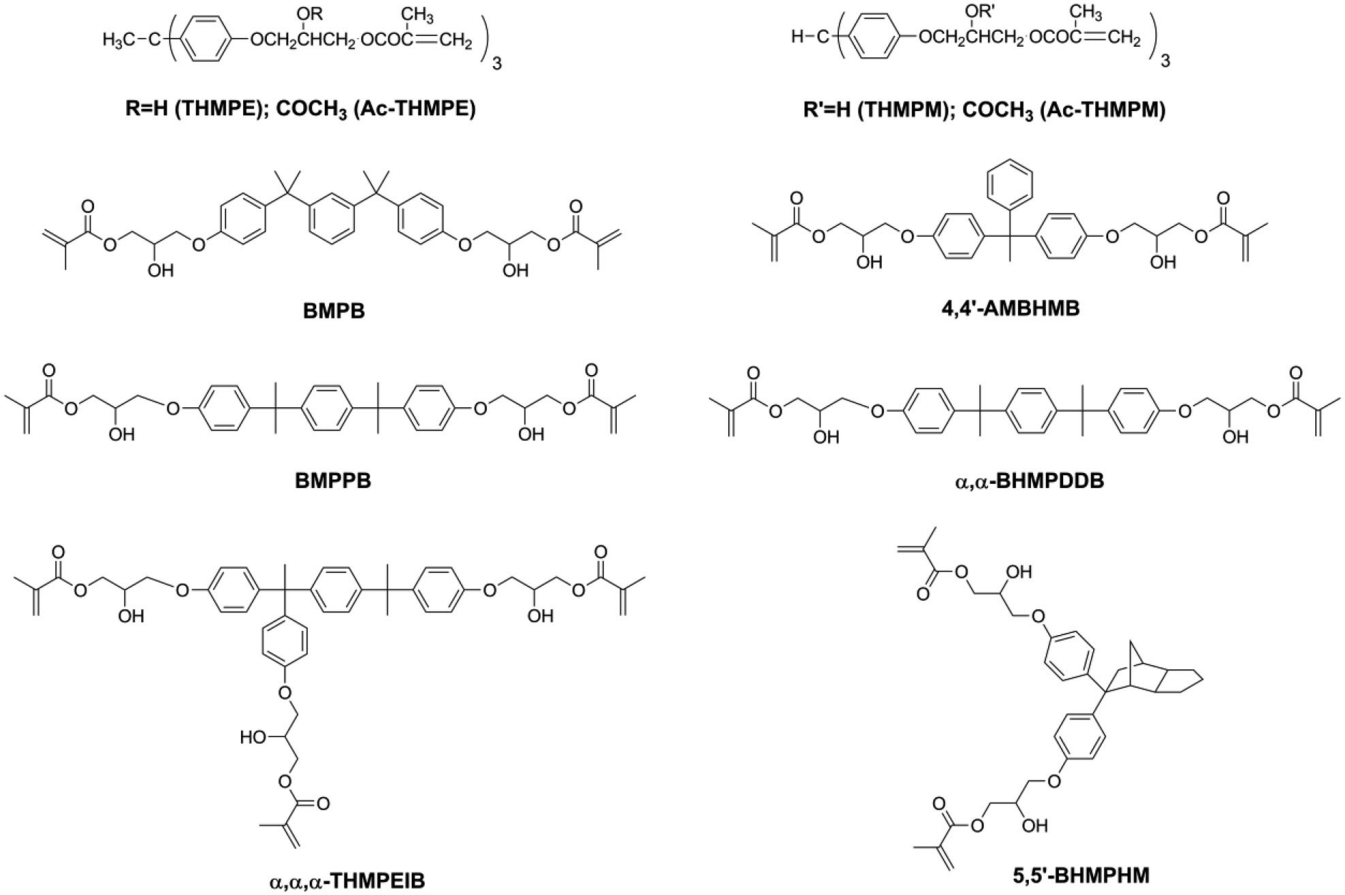
Structures of high molecular weight methacrylates for reducing polymerization shrinkage.

According to the composition and light curing conditions, DRCs exhibit volumetric shrinkage ranging from 1% to 6% [17]. The volumetric shrinkage of DRCs cannot be eliminated, but it can be reduced. As for the resin matrix, it is well known that volumetric shrinkage can be reduced by using monomers with a low reactive group (double bond) concentration, or high molecular weight monomers with the same amount of functional (double bond) groups [23–27]. Using mono-methacrylate is an effective way to reduce double bond concentration, but mono-methacrylate has no contribution to the cross-link density of the polymeric network which might influence the mechanical properties of DRC. Therefore, several methacrylate monomers with high molecular weight (as shown in Figure 1) have been synthesized and used to replace Bisphenol A glycidyl methacrylate (Bis-GMA) [28–35], which is the most used monomer in commercial DRCs. All these, newer monomers have been Bis-GMA analogues with more benzene or aliphatic rings in the structure. Due to these rigid groups, the use of these monomers led to reduced volumetric shrinkage of the resin matrix while maintaining mechanical properties, but also to a reduction in double bond conversion. However, filler loading, which also plays an important role for the properties of DRCs [36–38], may have to be lowered due to the high viscosity of these monomers.

**Figure 2.**
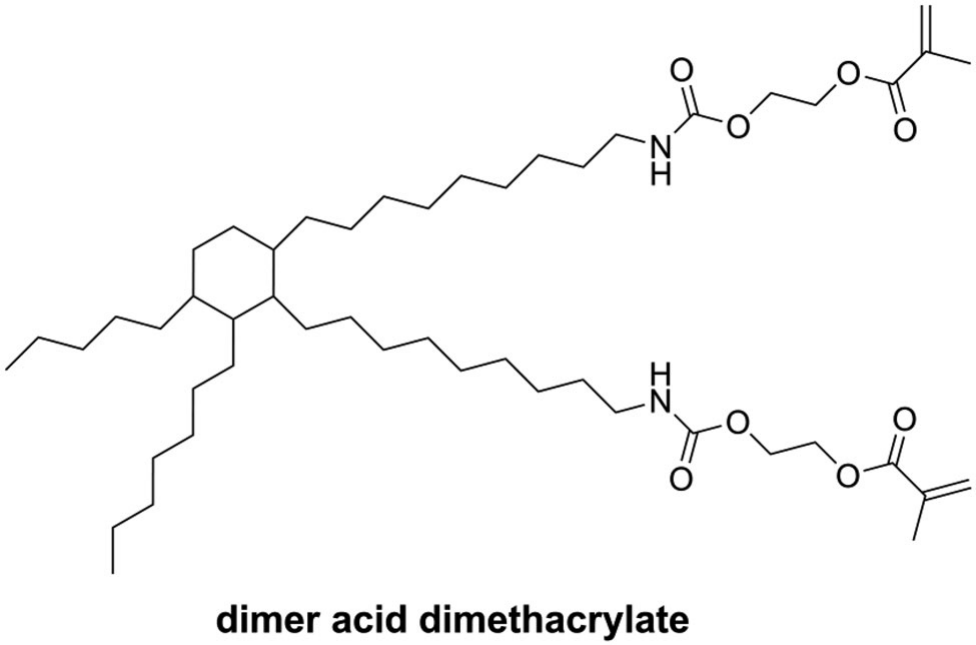
Structure of dimer acid dimethacrylate used in commercial dental resin composite.

Dimer acid dimethacrylate (as shown in Figure 2) is one kind of monomer with high molecular weight but low viscosity. In photopolymerization, dimer acid dimethacrylate showed a high degree of conversion coupled with low volumetric shrinkage and nearly negligible water sorption and is considered a good alternative to Bis-GMA [39]. NĭDurance is one kind of commercial DRCs containing dimer acid dimethacrylate, UDMA (bis-[(2-methacryloyloxy-ethoxycarbonyl)amino]-2,2,4-trimethylhexane), and Bis-EMA (Ethoxylated bisphenol A dimethacrylate). However, NĭDurance has been reported to have higher volumetric shrinkage than Bis-GMA-based commercial DRCs [40].

**Figure 3.**
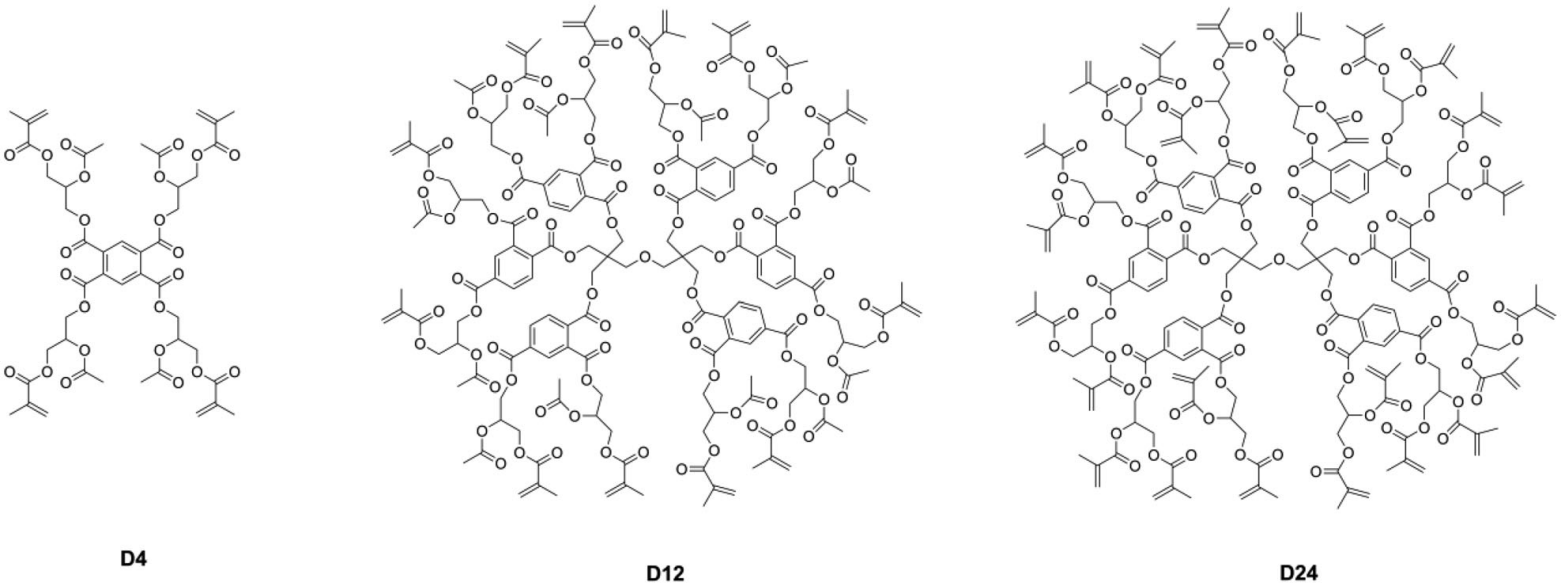
Structures of dendrimer macromers.

Different from linear monomers, dendritic macromers have a more compact structure [41] and less free volume [42] and can thus reduce the polymerization shrinkage of the resin matrix [43–45]. Viljanen et al. [46–50] prepared dendrimer macromers with 4 and up to 12 or even 24 methacrylate groups (as shown in Figure 3), but the high viscosity of these macromers became a problem for curing, and higher concentration of photoinitiation system was needed.

Compared with Bis-GMA (shrinkage 5.2%), the reactive diluent triethylene glycol dimethacrylate (TEGDMA, shrinkage 12.5%) has higher volumetric shrinkage [51]. Hence, developing a low-shrinkage diluent is also an effective way to achieve low-shrinkage DRCs.

**Figure 4.**
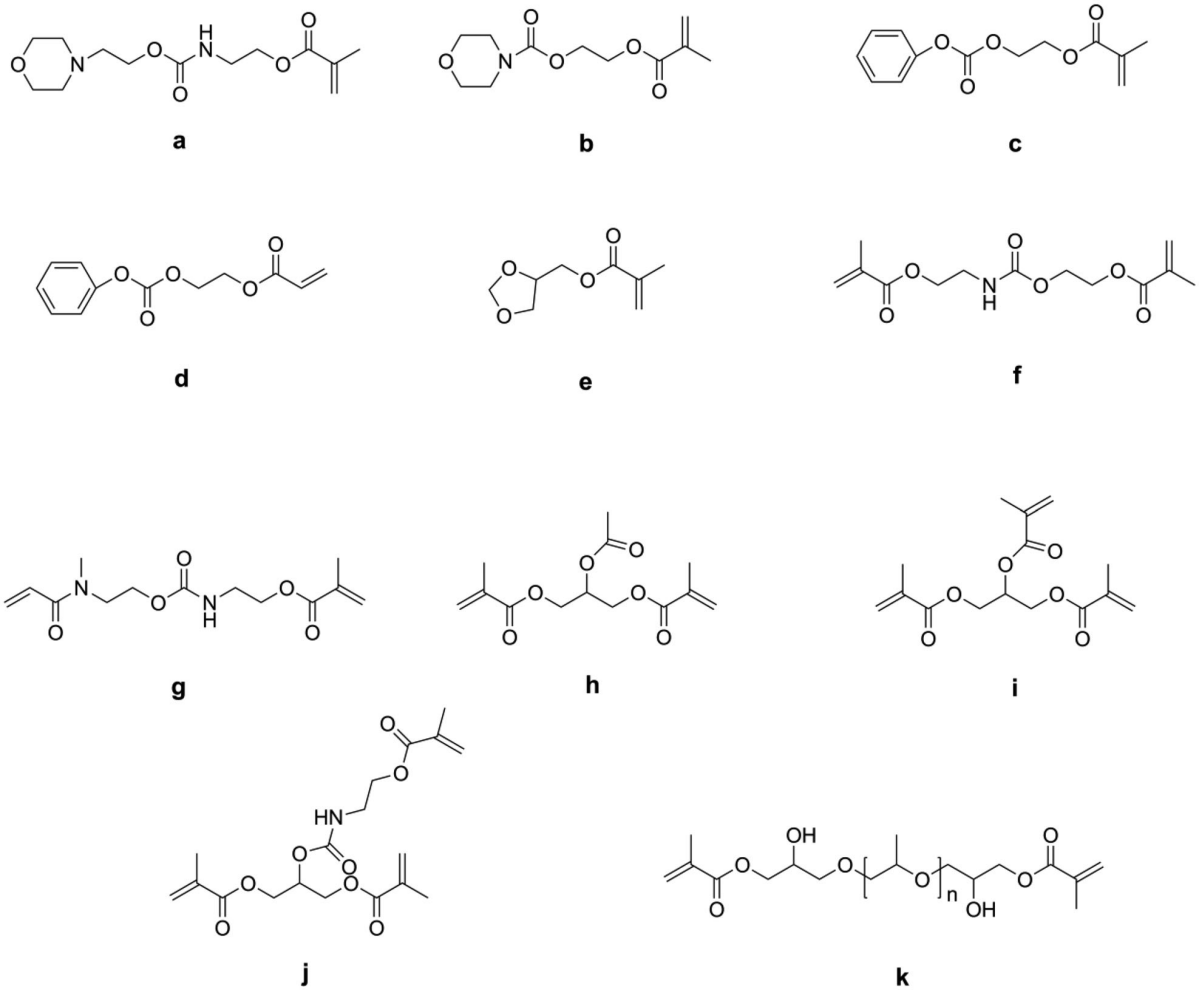
Structures of reactive diluents with low polymerization shrinkage.

Lu et al. [52] synthesized several mono-(meth)acrylates (as shown in Figure 4(a–e)) as alternatives to TEGDMA and mixed these with Bis-GMA to form dental resin systems. Due to their low double bond concentration, all these new resin systems had lower volumetric shrinkage than Bis-GMA/TEGDMA resin systems, but most of them had lower flexural strength and modulus because of the reduced cross-linking density.

Moszner et al. [53] synthesized five colorless and low-viscosity monomers (as shown in Figure 4(f–j)) and found they had lower volumetric shrinkage than TEGDMA and showed an acceptable performance as a diluent in Bis-GMA/UDMA-containing DRCs. Luo et al. [54] synthesized two methacrylate terminated oligomers named polyropylenglycol bis(2-hydroxy-3-methoxypropyl) dimethacrylates (PPMAs, as shown in Figure 4(k)) with different average molecular weights and used these to replace partially TEGDMA in Bis-GMA/TEGDMA resin systems. The results showed that when 20 wt.% of TEGDMA was replaced by PPMAs, the obtained DRCs exhibited significantly lower volumetric shrinkage. However, 10 wt.% of replacement had no effect, because the PPMA-containing DRCs were more homogeneous than the control DRC, which was not beneficial for reducing shrinkage [55].

### Ring-opening and cyclopolymerizable monomers

**Figure 5.**
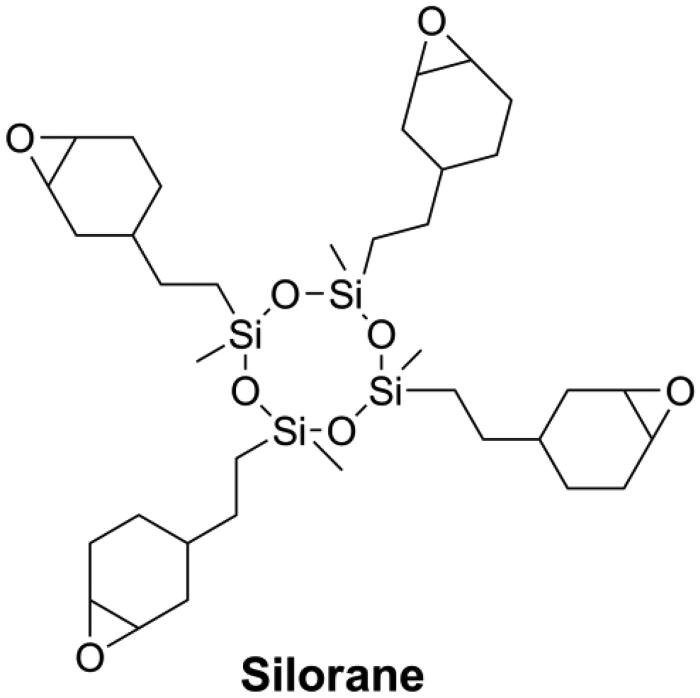
Structure of silorane used in commercial dental resin composite.

Besides reducing the double bond concentration of the resin matrix, several novel monomers that undergo ring-opening polymerization and cyclopolymerization were developed and introduced in dentistry to reduce polymerization shrinkage of DRCs. During ring-opening polymerization, when a bond that goes from a van der Waals distance to a covalent distance, another bond goes from a covalent distance to a near van der Waals distance, resulting in a certain extent of volumetric expansion, thus offsetting the shrinkage accompanying the chain propagation [56]. Cyclic monomers, such as epoxy [56–58], spiro orthocarbonates [59–62], oxetanes [63,64], and vinylcyclopropanes [21,65–67] can undergo ring-opening polymerization. Silorane (as shown in Figure 5) which undergo cationic ring-opening polymerization showed low shrinkage and good mechanical performance [68]. The commercial Silorane-based DRC named Filtek Silorane (3 M-ESPE, Seefeld, Germany) is the only methacrylate-free DRC that has a volumetric shrinkage less than 1% [69]. Nonetheless, Filtek Silorane had lower bond strength to Class-I cavity dentin than conventional methacrylate-based DRCs [70] and did not appear to offer any clinical advantages over methacrylate-based DRCs when used in the restoration of Class-II cavities [71].

**Figure 6.**
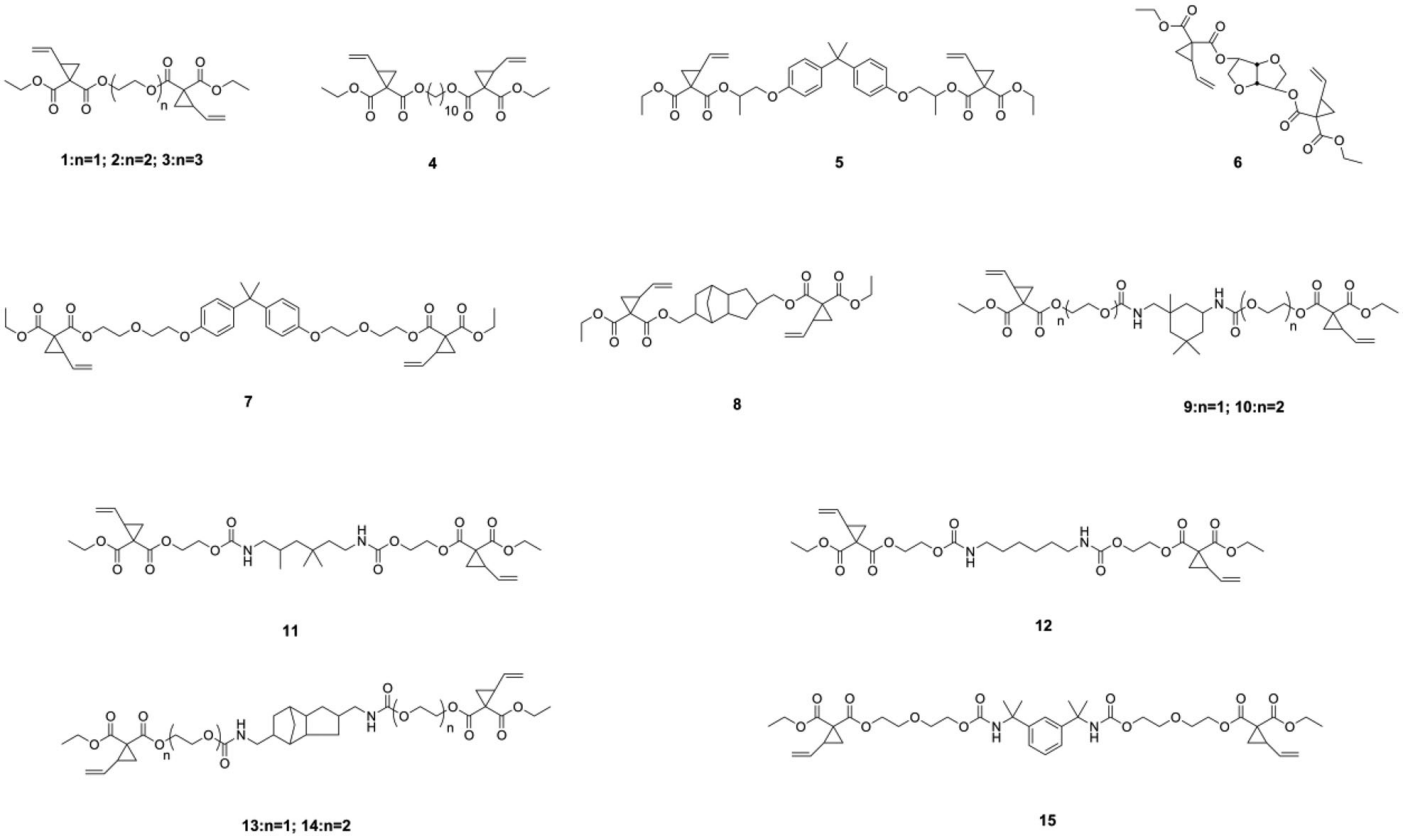
Structures of vinylcyclopropanes used in dentistry.

**Figure 7.**
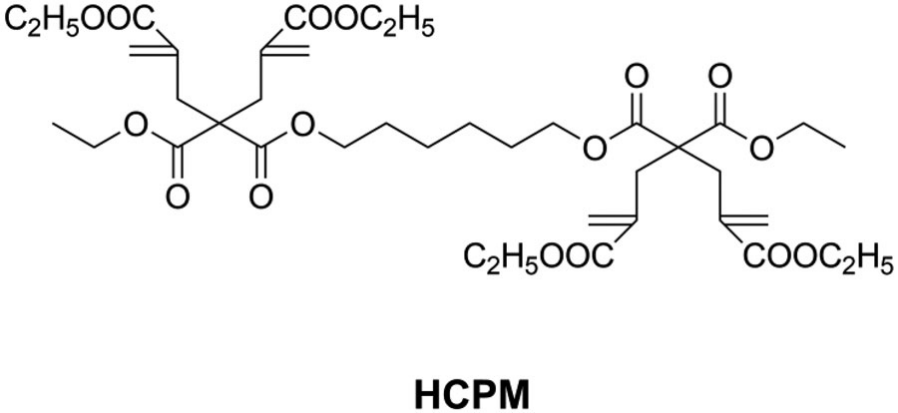
Structure of cyclopolymerizable monomer HCPM.

Compared with other cyclic monomers, vinylcyclopropanes are considered to have better resistance to humidity and acidic/basic impurities, which is essential for dental applications [21,66,67]. Catel et al. [66,67] and Tauscher et al. [21], synthesized a series of vinylcyclopropanes (as shown in Figure 6) to be used as a base resin and reactive diluents. After composition optimization, vinylcyclopropane-based DRCs showed promising mechanical properties and lower volumetric shrinkage than methacrylate-based DRCs. However, and unfortunately, it is difficult to access vinylcyclopropanes due to their inherent instability and the susceptibility of their double bond to engage in side reactions. Different from ring-opening monomers, cyclopolymerizable monomers can form aliphatic rings during polymerization, resulting in larger free volume and low shrinkage [72]. Peer et al. [73] synthesized a cyclopolymerizable dimalonate (HCPM, as shown in Figure 7) and concluded that it could be used as low shrinkage reactive diluent.

### Low shrinkage stress monomers

**Figure 8.**
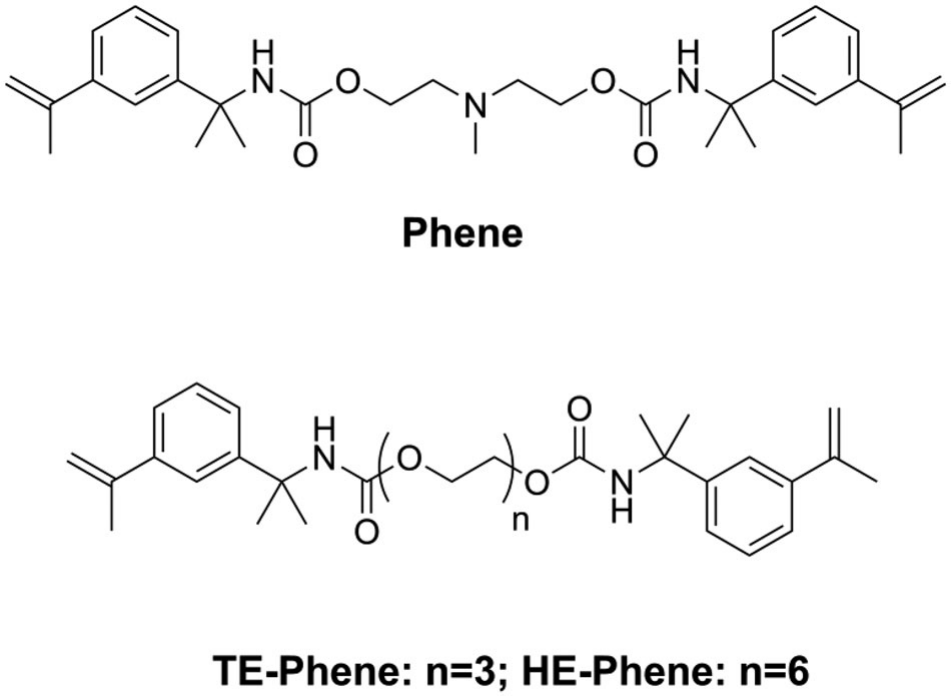
Structures of Phenes.

Though volumetric shrinkage is important for DRCs, dental restoration failures are mainly related to the development of shrinkage stress, which is a much more complex phenomenon than the volumetric change of the organic matrix. Shrinkage stress can be reduced through reducing volumetric shrinkage [22,54,74] and polymerization rate [75,76]. He et al. [77,78] synthesized three kinds of novel monomers (Phenes, as shown in Figure 8) containing an α-methylstyryl structure as a polymerizable group. Due to the resonance structure that delocalizes the double bonds, the α-methylstyryl group has a lower polymerization rate than the methacrylate group. Moreover, it was reported that polymerization of the α-methylstyryl group had an appreciable depropagation rate even at room temperature [79]. Both these effects could delay the vitrification state of the material, resulting in lower shrinkage stress.

**Figure 9.**
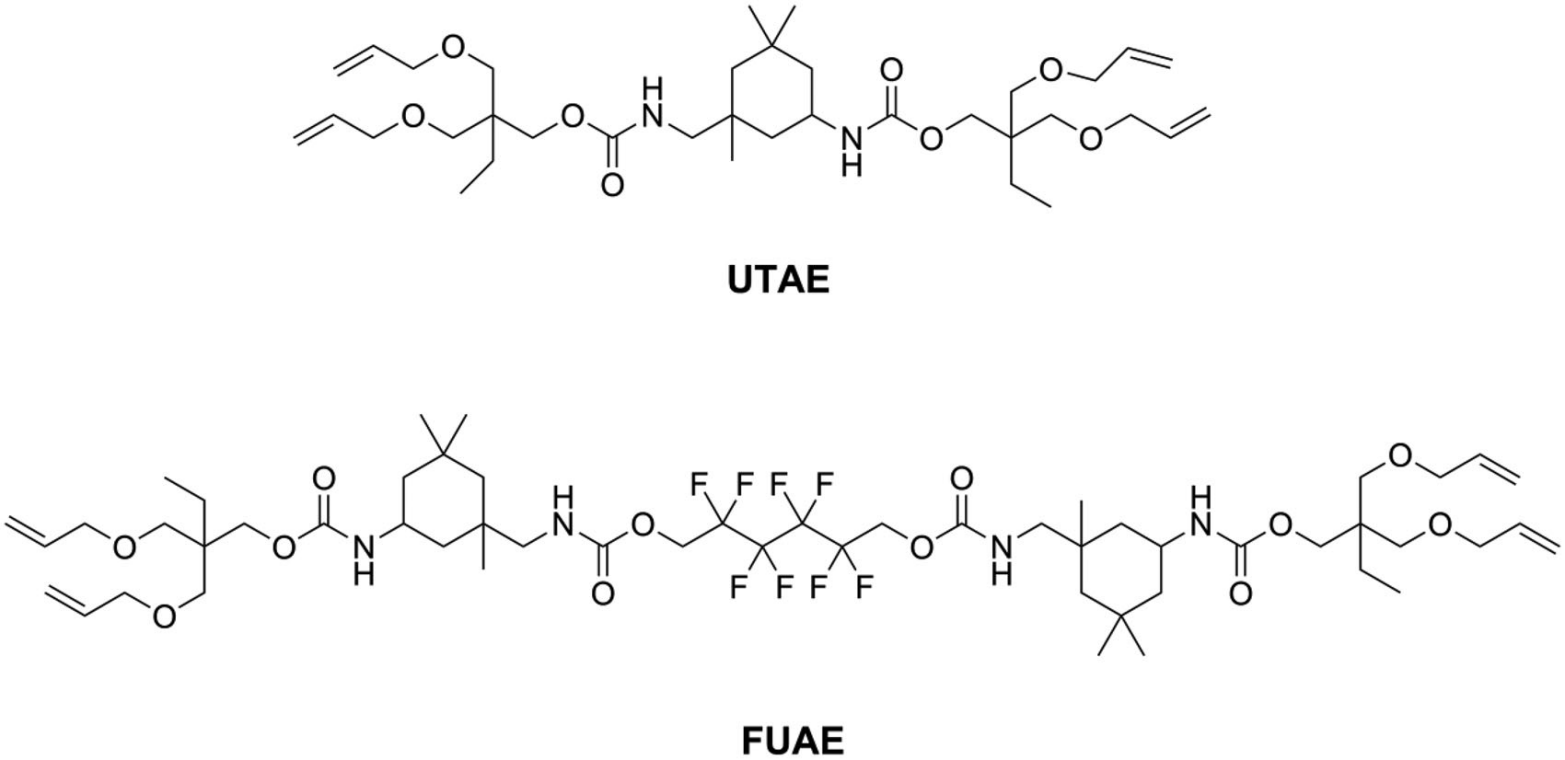
Structures of urethane-based allyl ethers.

Utilizing monomers which possess unique polymerization mechanisms is also a strategy for achieving low shrinkage stress and might be more effective than reducing volumetric shrinkage. The overall idea is delaying the gel point so that the stress can be released during polymerization. Different from the chain-growth mechanism of methacrylates, thiol-ene resin systems contained thiol and vinyl monomers, which polymerize *via* a step growth mechanism involving free-radical addition followed by a chain transfer reaction and were found to result in delayed gelation and low shrinkage stress [80–82]. However, since the thiol-ene resin systems showed lower flexural strength and modulus than commonly used Bis-GMA/TEGDMA resin systems [83], they were incorporated into a methacrylate resin system to form a thiol-ene-methacrylate ternary system [84]. To maintain flexural strength, there is a mass fraction limitation of thiol-ene resin systems in thiol-ene-methacrylate ternary systems. Beigi et al. [85] synthesized a urethane terta allyl ether monomer UTAE (as shown in Figure 9) and mixed it with commercial thiol pentaerythritol tetra (3-mercaptopropionate) (PETMA) to prepare a thiol-ene system. Subsequently, they added this thiol-ene system into a Bis-GMA/TEGDMA system and found that flexural strength and modulus of the ternary system was reduced significantly when the concentration of thiol-ene was higher than 20 wt.% in the resin. Using a synthesized fluorine-containing urethane-based allyl ether monomer FUAE (as shown in Figure 9), Fu et al. [86] were able to raise the amount of a thiol-ene system up to 30 wt.% in a ternary system without impairing flexural strength and modulus. The application of thiol-ene systems in the clinic is still rare, because of the distinct and unpleasant odor of thiol monomers, obvious even after increasing their molecular weight [87].

**Figure 10.**
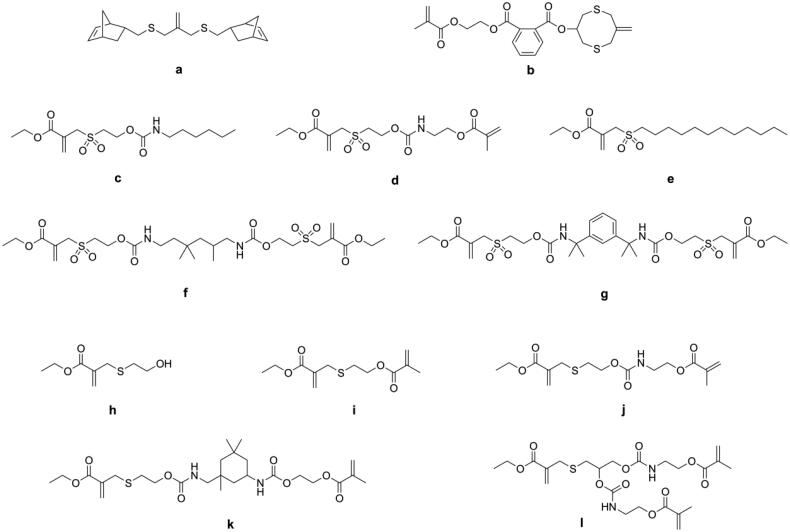
Structures of addition-fragmentation chain transfer monomers used in dentistry.

In recent years, covalent adaptable networks (CANs) have been used to reduce shrinkage stress. When subjected to specific stimuli, the covalent bonds in the network dynamically break, relax, and rearrange to form new bonds, thus allowing stress to be released during network formation [88]. Using addition-fragmentation chain transfer monomers to reduce the shrinkage stress of DRCs is a. type of CANs mechanism. Park et al. [89] incorporated allyl sulfide monomers (as shown in Figure 10(a,b)) into norbornene-methacrylate systems to obtain low shrinkage stress DRCs. The new DRCs showed more than 96% stress reduction compared with Bis-GMA/TEGDMA-based DRCs but also exhibited lower flexural strength. Lamparth et al. [90] and Crob et al. [91] evaluated allyl sulfone urethanes (as shown in Figure 10(c–g)) and allyl sulfide methacrylates (as shown in Figure 10(h–l)) as addition-fragmentation chain transfer agents for low shrinkage stress DRCs. The results revealed that all these agents could reduce shrinkage stress while maintaining flexural strength, but the maximum stress reduction was only around 36% at an agent concentration of 20 mol% in the resin matrix.

Considering the accessibility of raw material and overall properties of the final DRCs, using Phenes and addition-fragmentation chain transfer monomers seem to be the most promising approaches so far.

## Monomers as bactericides and anti-bacterial adhesion agents

In contrast to certain other restorative materials, DRCs have no intrinsic antibacterial properties, thus more biofilm accumulation occurs on the surface of DRCs, leading to the high incidence of secondary caries [92,93], which is one of the main reasons for clinical restoration failure [94]. Therefore, it is essential and necessary to endow DRCs with antibacterial properties. Generally, antibacterial properties can be classified as the ability to kill bacteria that surround or are attached to the surface of the DRCs and to inhibit bacteria adherence on the surface. Both these effects can be achieved by adding monomers with specific functional designs. Because the formation of biofilm is initiated by the adsorption of salivary proteins, it is expected that biofilm formation could be minimized by precluding protein adsorption [95].

### Bactericidal monomers

**Figure 11.**
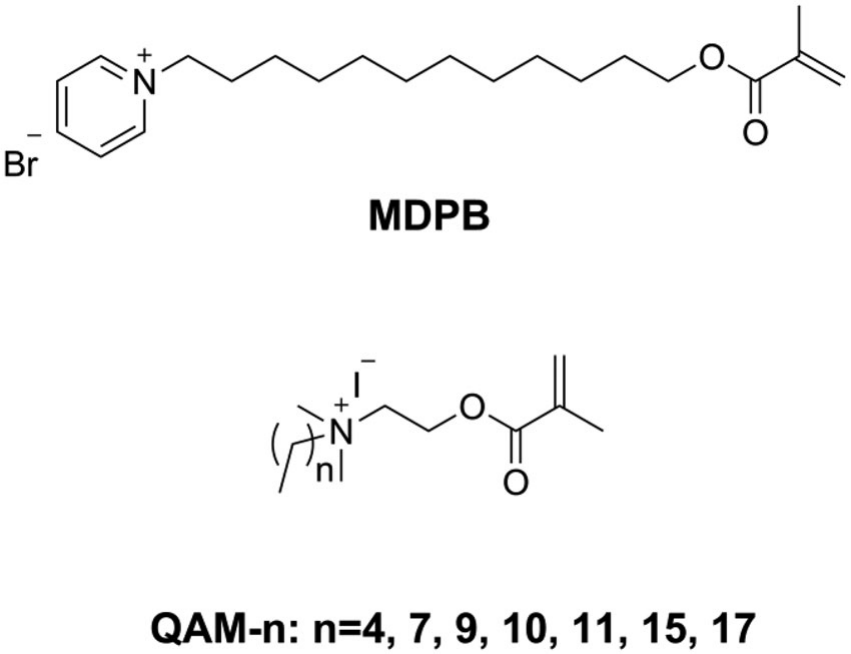
Structures of MDPB and QAM-n.

Releasable bactericides like chlorhexidine [96,97], benzalkonium chloride [98], and cetylpyridinium [99] can kill pathogens efficiently, but also have inevitable disadvantages, such as short-term effectiveness, impaired mechanical properties, and induced antibiotic resistance [100]. To achieve long-term antibacterial effectiveness, Imazato introduced a concept of ‘immobilized bactericide’ into dentistry [101]. An immobilizable bactericide is a monomer that contains a bacteriocidal group as well as a polymerizable group in its structure, which can then participate in the polymerization process and immobilize the bacteriocidal group in the polymeric network. The first immobilizable bactericide used in dentistry was methacryloyloxydodecyl pyrimidinium bromide (MDPB, as shown in Figure 11) invented by Imazato and co-workers [101–105]. Subsequently, several kinds of immobilized bactericides containing quaternary ammonium as the bacteriocidal group have been synthesized and used in dentistry.

He et al. [106] synthesized a series of quaternary ammonium mono-methacrylates (QAM-n, as shown in Figure 11) with an alkyl chain length ranging from 5 to 18 and found that antibacterial activity of the monomer against *Streptococcus mutans* increased when the alkyl chain length increased from 5 to 16 and decreased when the alkyl chain length increased further to 18, thus showing a typical cut-off effect. After incorporating 5 wt.% of QAM-n (n from 9 to 17) into Bis-GMA/TEGDMA resins, nearly all the obtained resins showed an inhibition effect on 6h young biofilm; only resins with QAM-15 and QAM-17 showed an inhibition effect on 24h mature biofilm, and all QAM-n had no negative effect on mechanical properties [107,108]. Because of a miscible problem with Bis-GMA/TEGDMA resin, 5 wt.% was the maximum mass fraction of QAM-n possible, which meant that the antibacterial activity of the prepared resins was not sufficiently strong. He et al. [109] used Bis-GMA, methyl methacrylate (MMA), and QAM-11 (DDMAI) to prepare resin systems with a high mass fraction (from 15 wt.% to 25 wt.%) and found the resin systems to show strong biofilm inhibitory effect, but also to have significantly lower flexural properties as well as higher water sorption and solubility.

**Figure 12.**
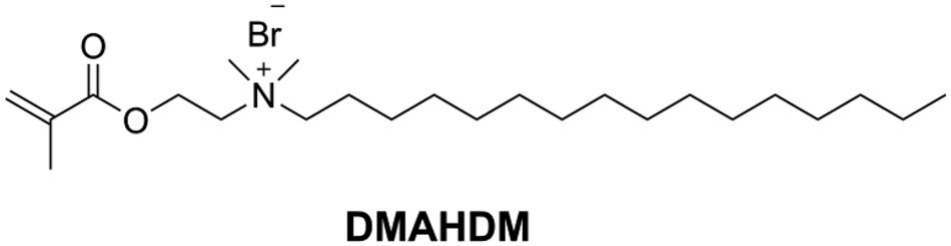
Structure of DMAHDM.

Dimethylaminohexadecyl methacrylate (DMAHDM, as shown in Figure 12) synthesized by the reaction between 2-(dimethylamino)ethyl methacrylate (DMAEMA) with 1-bromohexadecane is the most studied quaternary ammonium mono-methacrylate in recent years [110]. Wang [111] added 10 wt.% of DMAHDM into Bis-EMA/PMGDM (pyromellitic dianhydride glycerol dimethacrylate) resin matrix to prepare a DRC. The obtained DRC showed an excellent antibacterial rate (more than 90%) without compromising flexural properties. In another study [112] it was found that even 5 wt.% of DMAHDM in Bis-GMA/TEGDMA endowed the DRC with strong antibacterial activity while maintaining flexural properties and that the effect lasted for 6 months in distilled water. However, Cherchali et al. [113] reported that a DRC with 10 wt.% of DMAHDM in the resin matrix (Bis-GMA/TEGDMA) had good antibacterial activity but lower flexural strength than the control DRC. Vidal et al. [114] also reported that 5 wt.% and 10 wt.% of DMAHDM in DRCs did not influence mechanical properties in dry condition. However, after 30 d of water immersion, the mechanical properties of the DMAHDM containing DRCs were lower than those of the control DRC. The different results in the above-mentioned studies might be due to the differences in matrix composition, filler characteristics and filler content.

**Figure 13.**
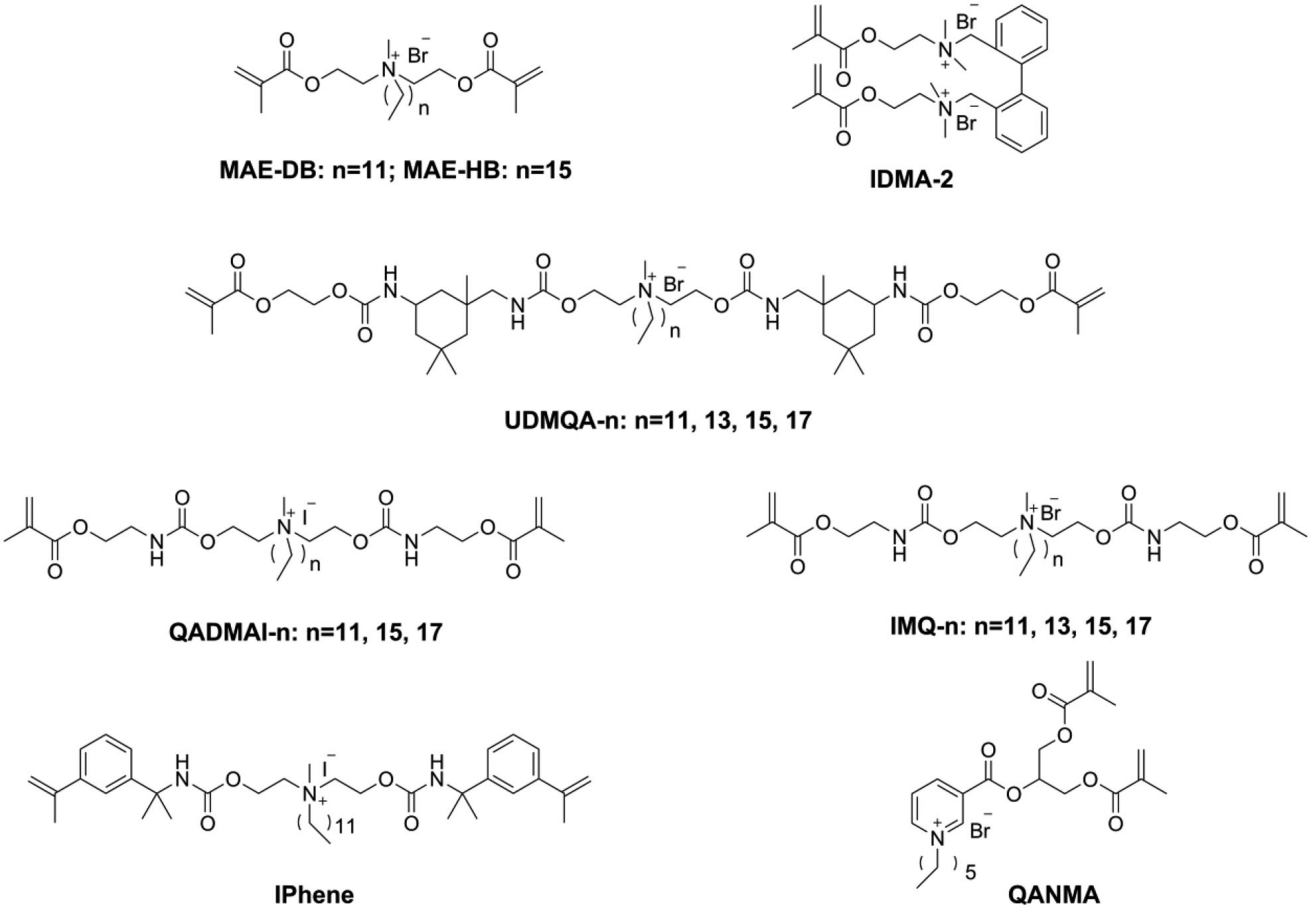
Structures of quaternary ammonium with di-polymerizable groups.

Addressing the issues of poor miscibility and lack of contribution to cross-linking density of quaternary ammonium mono-methacrylate, quaternary ammonium monomers with multi-polymerizable groups (as shown in Figure 13) were designed and synthesized [115,116]. However, due to the reduced quaternary ammonium concentration in the structure, a higher mass fraction (from 10 wt.% to 50 wt.%) was needed to guarantee sufficient antibacterial activity of these monomers, lowering the mechanical properties [117–122]. Therefore, further optimization work is needed for these monomers.

**Figure 14.**
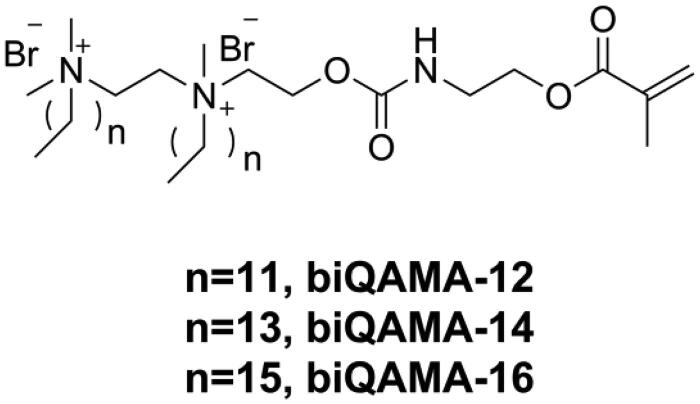
Structures of bi-quaternary ammonium mono-methacrylates biQAMAs.

Because the antibacterial activity of DRCs is dependent on the concentration of the incorporated quaternary ammonium structure, using a monomer with high quaternary ammonium concentration might endow DRCs with strong antibacterial activity at a low-dose drug loading. Bis-quaternary ammonium monomers with a higher concentration of quaternary ammonium structure might have the potential to provide sufficient antibacterial activity even at a low molar concentration [123]. He et al. [124] designed three kinds of bi-quaternary ammonium mono-methacrylates (biQAMAs, as shown in Figure 14) and added 5 wt.% of these into Bis-GMA/TEGDMA resins to prepare DRCs. The results showed that all biQAMA-containing DRCs exhibited strong antibacterial activity (more than 90% antibacterial rate against *S. mutans*) and comparable physicochemical properties as the control DRC, except that the water sorption of biQAMA-containing DRCs was higher than that of the control. However, only biQAMA with a side alkyl chain length of 12 had no influence on the cytotoxicity of the DRC, whereas the other two biQAMAs increased the cytotoxicity of the DRC.

**Figure 15.**
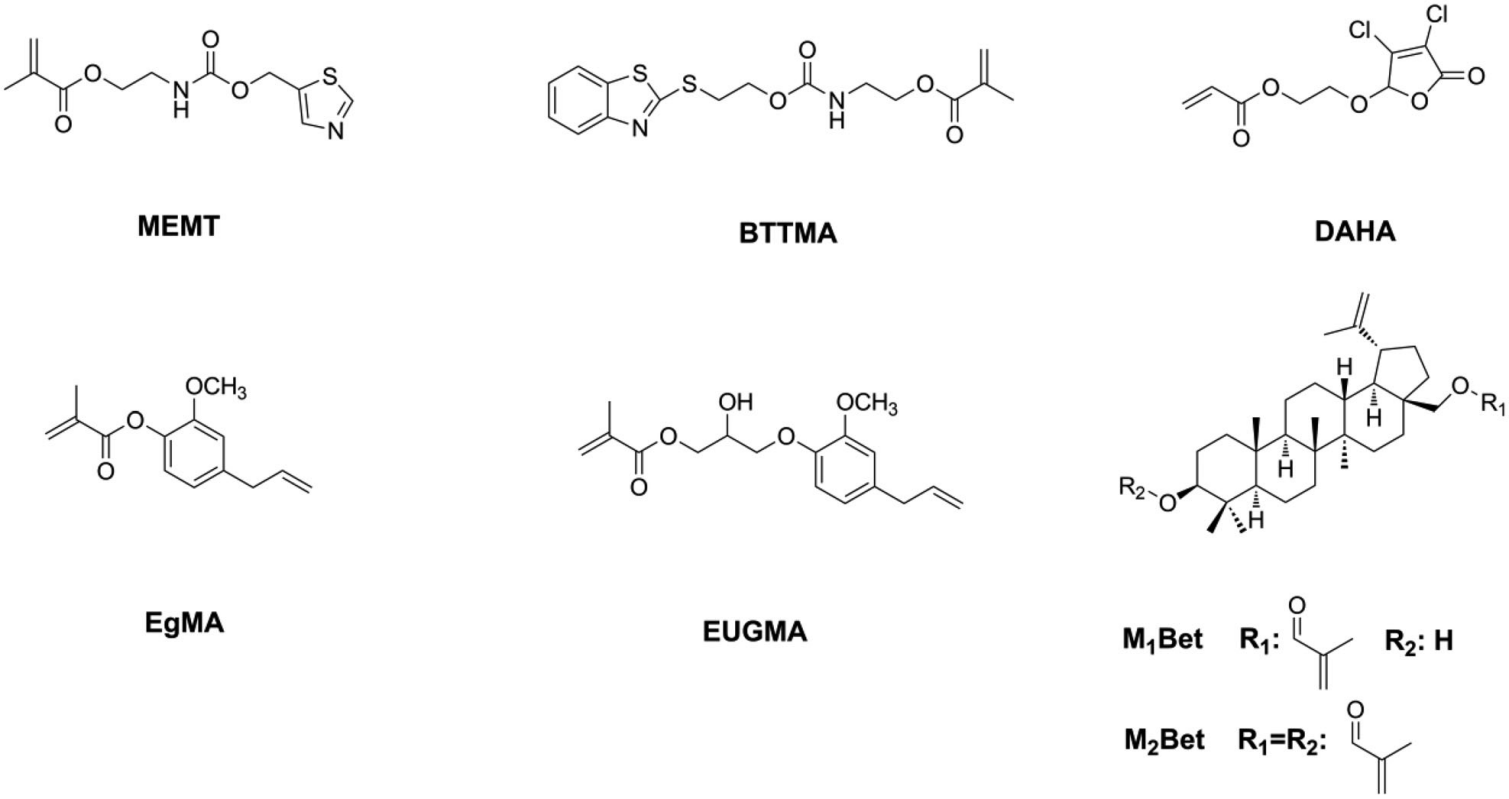
Structures of non-quaternary ammonium immobilized bactericides.

Although the quaternary ammonium group has strong and broad-spectrum antibacterial activity, its hydrophilic structure can increase the water sorption of DRCs and influence the water resistance of DRCs. Moreover, the antibacterial activity of quaternary ammonium methacrylate-containing DRCs can be reduced due to the electrostatic interactions between quaternary ammonium salts and proteins in saliva [125,126]. Therefore, other bactericidal structures, such as heterocyclic rings [127–129] and natural bactericidal structures [130–135], were chosen to be connected with the (meth)acrylate group and then applied in dentistry as immobilizable bactericides (as shown in Figure 15). All these monomers endowed DRCs with antibacterial activity without sacrificing physicochemical properties [127–135], thus showing great potential for clinical applications.

All the above studies focused on how to kill bacteria effectively without considering the influence of the bactericides on the oral microbiome. However, as an unbalanced oral microbiome may be detrimental to general health [136], developing antibacterial agents that are friendly to the commensal microbiome should be the aim of future research.

### Anti-bacterial adhesion monomers

**Figure 16.**
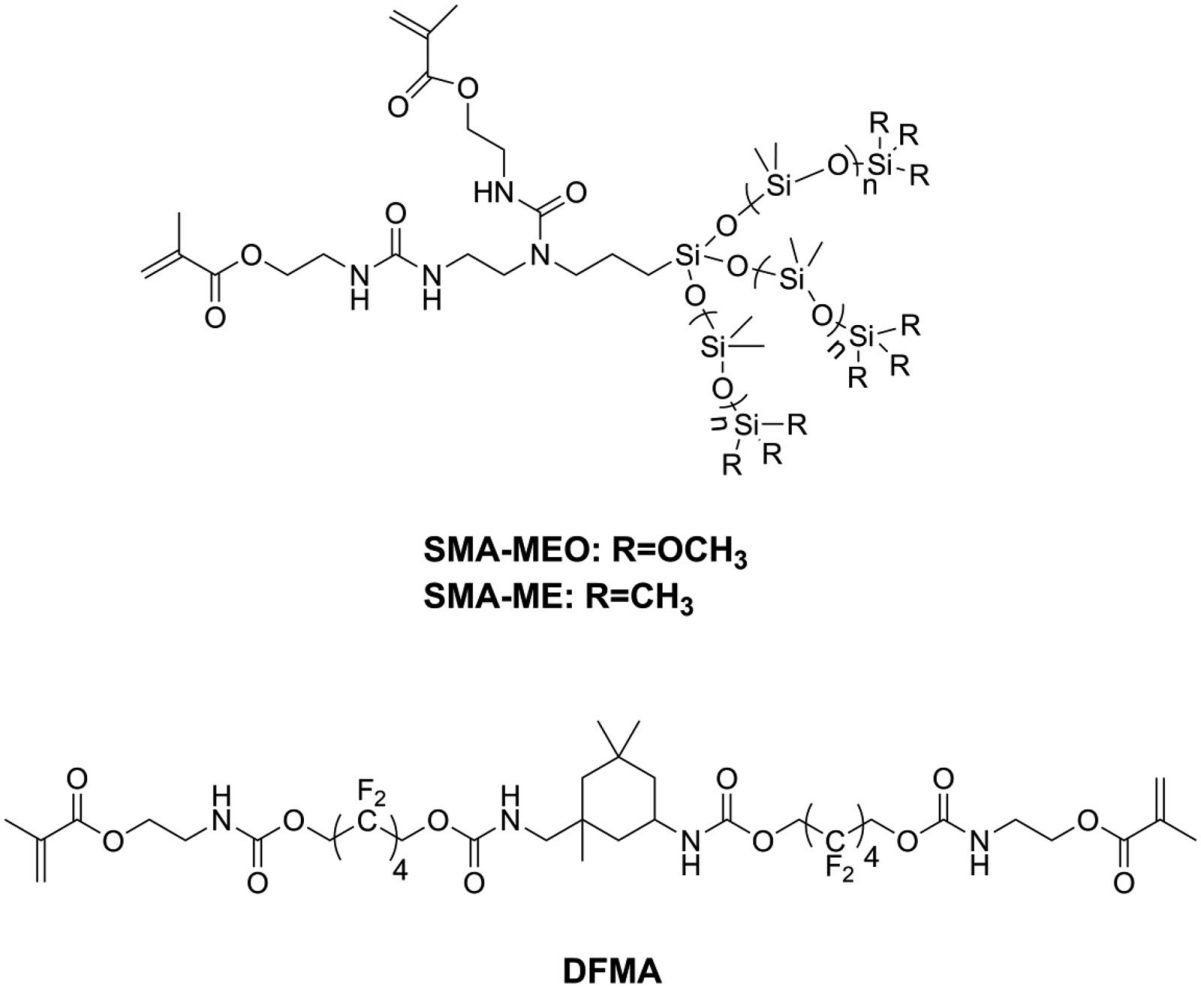
Structures of methacrylates for endowing dental resin composites with antibacterial adhesion activity.

Bacterial adhesion is reversable and considered to be the most important step in biofilm formation [137]. Thus, modification of DRCs to be anti-adhesive against cariogenic bacteria should be an effective way to reduce biofilm formation. According to surface thermodynamics, bacterial adhesion will not be favored if it causes the free energy function to increase [138]. Under salivary conditions, it is considered that bacteria with high surface free energy have difficulty in accumulating on surfaces with low surface free energy. Because bacteria in early plaque such as *S. mutans*, *Streptococcus sanguis*, and *V. parvula* are all high surface energy bacteria [139,140], modifying DRCs to have low surface energy might be beneficial in preventing secondary caries. Silicon-based materials is one kind of materials with low surface free energy and have been widely used in antifouling coating [141,142]. Tong et al. [143] prepared two kinds of silicone methacrylates (SMA-ME and SMA-MEO, as shown in Figure 16) and incorporated these into Bis-GMA/TEGDMA. The obtained resins did not only display an anti-bacterial adhesion effect but also resistance to protein adsorption, due to decreased surface free energy and increased hydrophobicity. However, silicone methacrylates displayed significantly lower flexural strength and modulus. Experimental silicone methacrylate-containing DRCs may have promising mechanical properties, reduced biofilm formation, acceptable cytotoxicity, and good bond strength to adhesive-treated dentin [144,145]. Fluorinated polymers are typical materials with low surface free energy and are attractive for dental application due to their advantages such as good biocompatibility, resistance to a wide range of chemicals, and potential resistance to bacterial adhesion [3]. Zhang et al. [146] synthesized a fluorine-containing methacrylate (DFMA, as shown in Figure 16) and used it to replace Bis-GMA as a base resin in DRCs. It was found that DFMA-based DRCs exhibited anti-bacterial adhesion effects, resistance to protein adsorption as well as better physicochemical properties than a Bis-GMA-based DRC.

**Figure 17.**
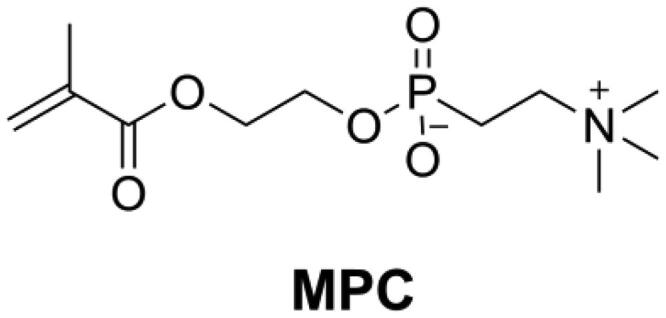
Structure of MPC.

2-Methacryloyloxyethyl phosphorylcholine (MPC, as shown in Figure 17) is a monomer that has an excellent ability to repel protein adsorption and prevent bacterial adhesion. Because of the high hydrophilicity of MPC, there would be an abundance of free water on the surface of MPC-containing polymers that can detach proteins effectively [147,148]. Incorporation of MPC into DRCs reduced protein adsorption and bacteria attachment on the surface of DRCs [148]. The combined use of antibacterial monomer quaternary ammonium dimethylaminohexadecyl methacrylate (DMAHDM) and MPC, led to a DRC with a strong biofilm inhibitory effect [149]. However, the concentration of MPC in DRCs should be optimized, because too high an MPC concentration will reduce mechanical properties and increase water sorption.

## Monomers as radio-opaque agents

Radio-opacity is an essential property for DRCs, allowing restoration to be easily distinguished from tooth tissue through X-ray examination. This distinction is beneficial for precisely localizing the interface between restoration and tooth and for assessing the clinical effectiveness of the restoration [150]. It is also worth noting that too high a radio-opacity may cause problems in novel medical imaging systems such as cone-beam x-ray system and magnetic resonance imaging [151,152].

**Figure 18.**
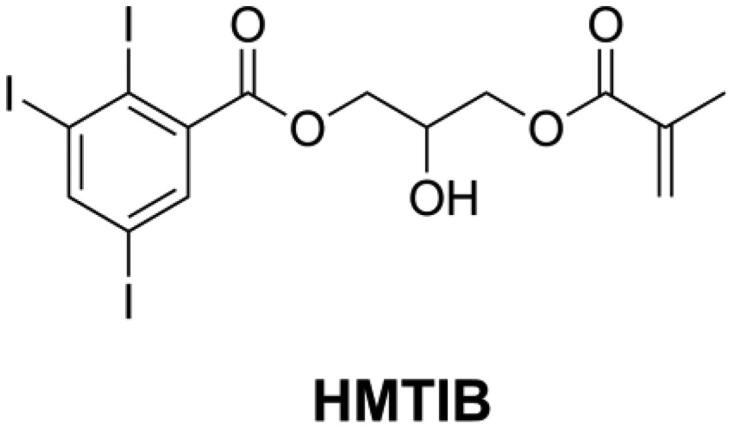
Structure of radio-opaque methacrylate HMTIB.

Usually, the radio-opacity of DRCs is dependent on the type and amount of inorganic fillers. However, some specific DRCs like flowable DRCs and E-glass fiber reinforced DRCs may have insufficient radio-opacity [153,154] because of their limited filler loading. For this reason, it is necessary to increase the radio-opacity of the resin matrix. The attenuation of X-rays in materials is associated with the density of the materials and with the atomic number and electron density of the elements that constitute the materials. Atoms with high atomic number can cause significant attenuation of X-rays [155]. Iodine is one kind of high atomic number atom with high electronic density and can thus block X-rays effectively. He et al. [107,109,118,120] synthesized several kinds of quaternary ammonium methacrylate with iodine anion and found that all these monomers endow dental resins with both antibacterial activity and radio-opacity. However, these monomers had a low concentration of iodine anion and therefore limited capacity of improving radio-opacity. He et al. [156] synthesized a monomer named 2-hydroxy-3-methacryloyloxypropyl- (2,3,5-triiodobenzoate) (HMTIB, as shown in Figure 18) with a high concentration of iodine element and mixed with Bis-GMA and MMA to prepare E-glass fiber reinforced composites (EFRCs). The results showed that HMTIB increased the radio-opacity of EFRCs significantly, and the obtained EFRCs had higher flexural strength, higher modulus, and lower water sorption.

## Monomers as Bis-GMA substituents

**Figure 19.**
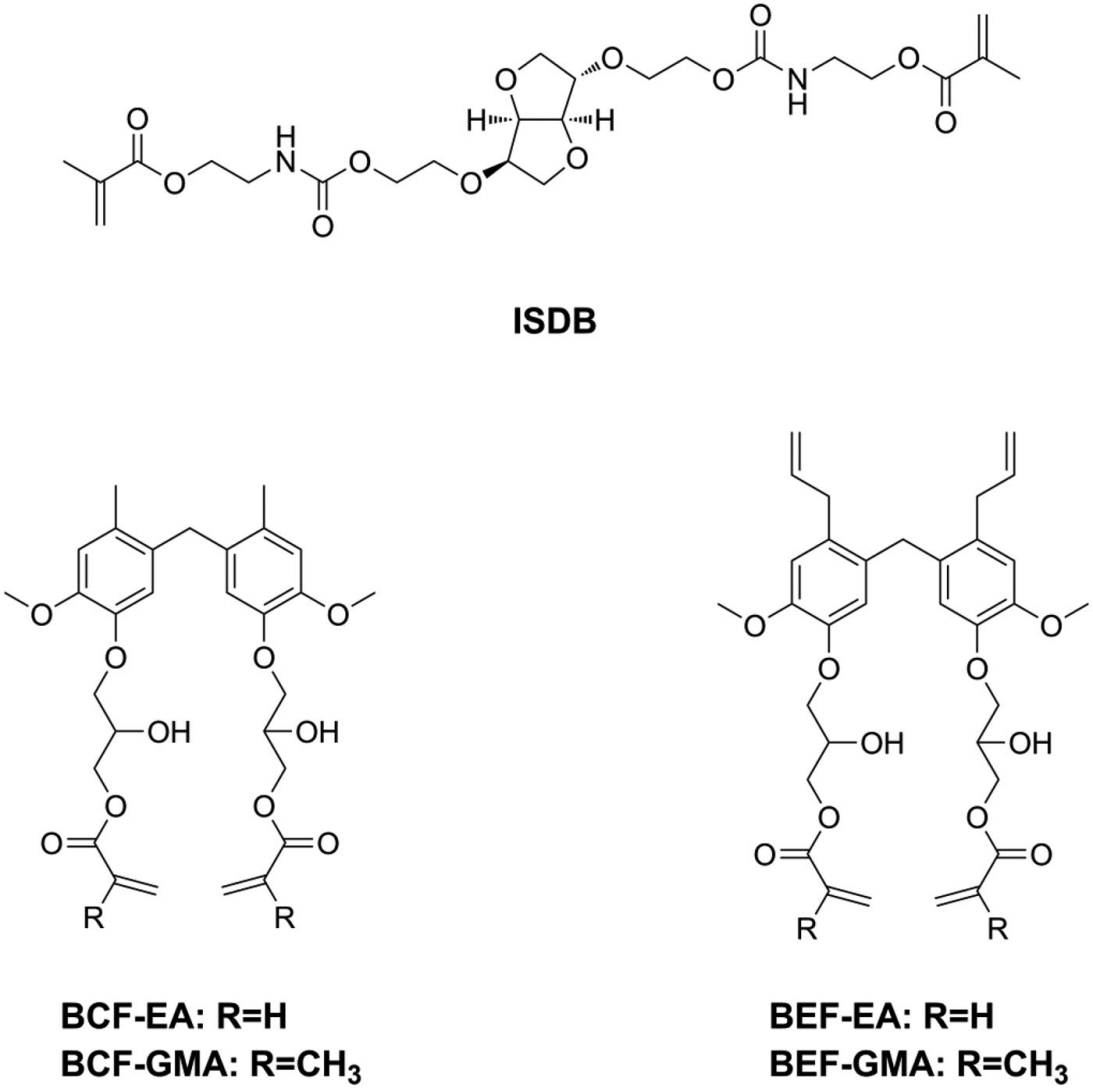
Structures of monomers with no estrogenicity.

Since being invented, Bis-GMA, which is a derivative of Bisphenol A (BPA), have dominated as the main monomer in DRCs. BPA is an endocrine-disrupting compound that can cause several diseases [157–161]. Although BPA is not a component of dental materials, and although alternative synthetic routes of Bis-GMA no longer require BPA, several studies still found the presence of BPA in patients’ urine and saliva after dental procedures [162]. Moreover, the latest studies showed that BPA may be released during the grinding and degradation of dental materials [163,164]. Consequently, numerous BPA-free monomers have been developed and used as alternatives to Bis-GMA, and most of them have shown promising properties and great potential in dentistry [74,146,165–177]. However, the estrogenicity of these new developed BPA-free monomers has not been investigated. Jun et al. [178] used isosorbide to synthesize light polymerizable isosorbide-derived biomonomers (ISDBs, as shown in Figure 19) and prepared Bis-GMA free sealants with ISDBs. They found the ISD-based sealants to have properties comparable to a Bis-GMA-based sealant and no estrogenicity. Sun et al. [179,180] tested the estrogenicity of two bio-based phenols and used these two phenols to prepare a series of BPA-free monomers (as shown in Figure 19), after confirming the absence of estrogenicity. Subsequently, they used these monomers to prepare Bis-GMA-free DRCs. Though these Bis-GMA-free DRCs exhibited acceptable physicochemical properties, certain properties still need to be improved to maintain the same level of performance as Bis-GMA-based DRCs.

## Conclusion

Tailoring monomers is an effective way of endowing dental resin composites with specific functions that prolong the service life of the restorations and reduce their potential harm to patients. However, so far, improving some properties has inevitably led to the impairment of other properties. Therefore, greater efforts should be made to develop novel monomers or to optimize the composition using existing monomers for the purpose of achieving low shrinkage and shrinkage stress, inhibited biofilm formation, resistance to enzymatic and hydrolytic degradation, or remineralization.
